# A method for IoT devices test case generation using language models

**DOI:** 10.1016/j.mex.2025.103340

**Published:** 2025-04-28

**Authors:** Sumit Kumar, Kiran Napte, Ruchi Rani, Sanjeev Kumar Pippal

**Affiliations:** a Symbiosis Institute of Technology, Pune Campus, Symbiosis International (Deemed University), Pune, Maharashtra 412115, India; b Department of Electronics and Telecommunication, Pimpri Chinchwad College of Engineering and Research, Pune, Maharashtra 412101, India; c Department of Computer Engineering and Technology, School of Computer Science and Engineering, Dr.Vishwanath Karad MIT World Peace University, Pune, Maharashtra 411038, India; d Department of CSEAI, GL Bajaj Institute of Technology and Management, Greater Noida, Uttar Pradesh 201310, India

**Keywords:** IoT, Testing, Language models, NLP techniques, Test Case Generation, Automated Test Case Generation in IoT Devices Using Language Models

## Abstract

The rapid growth of IoT and electronic systems has led to complex real-time data processing and management solutions. However, these systems present significant software and hardware testing challenges, often requiring manual, time-consuming testing efforts. To address this, an automated end-to-end testing framework is essential for improving efficiency and reliability in IoT system development. With advancements in Natural Language Processing (NLP) and language models, automated test case generation systems can now create structured test cases in programming languages while ensuring code integrity and style. Applying these techniques to IoT projects streamlines testing, enhances accuracy, and reduces workload. This paper introduces TCG-IoT (Test Case Generation for IoT Systems), an automated testing framework designed to generate comprehensive test cases and actionable event lists based on technical and data specifications. Unlike existing frameworks that depend on model-based testing or simulation environments, TCG-IoT uniquely integrates a Retrieval-Augmented Generation (RAG) mechanism, a vector knowledge base of IoT standards, and code generation via Code-Llama to directly produce structured, executable C-code scripts for software and manual steps for hardware components. The results demonstrate that TCG-IoT delivers high-quality, context-aware test cases and scripts with maximum system coverage, ensuring secure, efficient, and scalable IoT development.

• Automates test case generation for IoT hardware and software components.
• Enhances test coverage and reliability using language models.
• Evaluates performance through case studies on smart home automation.

## Graphical abstract

**Figure:**
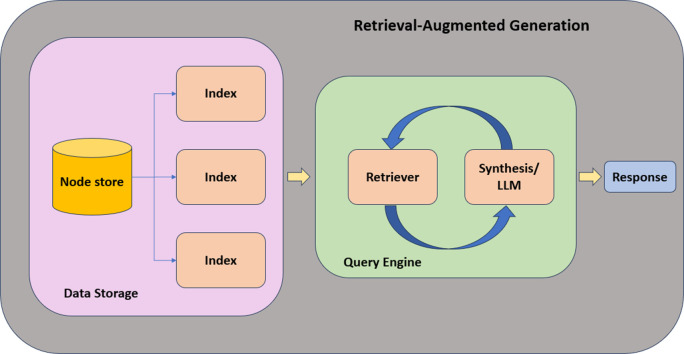

Specifications table

**Table:** 

Subject area:	Machine Learning, Deep Learning, Software Testing, IoT Security
More specific subject area:	Test Case Generation, IoT Device Security, Language Models, Automation in Software Testing
Name of your method:	Automated Test Case Generation in IoT Devices Using Language Models
Name and reference of original method:	NA
Resource availability:	Python, TensorFlow/PyTorch, OpenAI GPT, Google Colab, Local Machines

## Background

IoT (Internet of Things) is a network of interconnected physical devices embedded with sensors, software, and communication capabilities that enable data collection, sharing, and remote control. The Internet of Things (IoT) has taken off in recent years, and smart devices are expected to transform how we live, work, and interact with technology. These embedded systems can be technically complex since they process and manage huge chunks of information in real-time. In recent years, governments, industry, and academia have made much progress in developing such systems. Since these systems are inherently complex and are known to process huge bands of data quickly, testing these systems effectively becomes crucial to ensure smooth operation and quality results. IoT testing presents challenges both in the hardware and software domains. Natural Language Processing (NLP) is a subfield of artificial intelligence that enables machines to understand, interpret, and generate human language. In IoT testing, NLP extracts structured test requirements and generates test cases from unstructured technical documents or user prompts. Large Language Models (LLMs) are deep learning models trained on massive text datasets to perform language understanding and generation tasks. In this work, LLMs like Llama 2 and Code Llama automatically generate test cases and scripts from contextual queries. With the advent of NLP techniques and language models, researchers and engineers have been working on developing frameworks for software testing. These frameworks specialize in writing software test cases in a certain programming language based on the technical specs provided. These frameworks mimic project-specific details like code style, design principles, etc. [1]. One of the major use cases of language models is understanding the context and structure within the data. This paper, based on this principle, aims to employ open-source language models to propose an automated testing framework, TCG-IoT (test case generation for IoT systems), for IoT devices with a special focus on both the hardware and software components of the system, which can serve as an improvement over the current tools for testing IoT systems [2]. This work aims to generate a comprehensive suite of testing requirements, test cases, and scripts for IoT testing. The proposed methodology involves selecting an appropriate open-source language model. It uses techniques like fine-tuning and retrieval-augmented generation (RAG) to customize its outputs for IoT testing and generate results that maximize coverage and ensure system reliability. Retrieval-Augmented Generation (RAG) is a hybrid approach that combines document retrieval with generative language models. It enhances the model’s ability to produce accurate, context-aware outputs by retrieving relevant knowledge from a vector database and then using it to generate meaningful responses. A general technical specification for an IoT project contains a project overview, which is a brief description of the project, a description of the system architecture detailing the hardware and software components, technical requirements, hardware and software specifications, details on the data flow, and processing including the data storage solutions as well specification of communication protocols between devices, gateways and the central IoT platform. Dealing with such technically dense documents and generating test case events requires the model to understand context, the underlying relationship between the entities, and parsing structured content. Industry and academia have found that language models such as GPT [3], Llama [4], Alpaca [5], etc., fulfill this criterion and perform well as per various evaluation benchmarks. Jessica et al. [6] compare language models' performance and reasoning ability. This paper explores various language models, their tendencies, ease of deployment, and how well the model can handle technical content. As machine learning becomes more democratized by the day, there can be seen a noticeable rise in open source language models backed by big tech organizations and developer communities such as Llama (large language model released by Meta), Mistral, Dolly, Falcon, etc. This allows researchers and engineers to quickly get their ideas off the ground and develop applications suited for various industries that can solve many problems. This work proposes a generalized testing framework for IoT systems by deriving inspiration from software development's V-Model (Verification and Validation Model), which states that the appropriate testing requirements should be defined first. The generated test cases should be designed and implemented. Hynninen et al. detailed the alignment of software testing phases to V-Model Phases in the paper [7]. In addition, the model emphasizes validation and verification of the outputs at every stage. The generated test cases must be compared against the formal specifications provided, as seen in [8]. This work discusses in detail the intersection of software and IoT testing and how design principles such as the V-Model can be implemented to formulate life cycles for both these domains. One of the primary use cases of the proposed framework is allowing the system engineer, designer, or manufacturer to understand the various requirements, performance criteria, and feasibility of the system by generating a comprehensive suite of real-world test case scenarios that follow the design principles and best practices in the industry as discussed in [9], even before the hardware acquisition and assembly stage. This can save both time and resources for the designated engineer, proving to be a meaningful automation. The work emphasizes considering the best IoT testing practices utilized in the industry. It achieves that by collecting meaningful literature in this domain and then using querying and retrieval techniques via language models on the literature corpus by the set of testing requirements and technical specifications given by the user. The framework achieves that by implementing RAG (Retrieval Augmented Generation) [10], an AI framework for acquiring facts from an external knowledge base to supplement language models to give users the most accurate and up-to-date insights. This constructed knowledge facilitates a series of operations within the framework to ensure that the test cases generated are based on useful principles and are more focused on testing IoT systems. There are no fixed metrics to evaluate the results of the testing framework. However, the test cases generated can be scored based on testing criteria. Since language models can be adaptive and provide users with added feasibility, these testing criteria can also be defined by the user and given as instructions to the model through effective, prompt engineering [11]. In the proposed work, a pre-defined set of testing criteria has been established beforehand, and the generated test cases are evaluated based on how well they comply with the defined criteria.

IoT systems manage huge bandwidths of data in real time. Hence, ensuring adequate testing of these systems for reliability and quality results is crucial. With the advent of complex NLP techniques and language models, we have seen testing frameworks being developed for software where models can generate code and write tests for software projects. However, automated test case generation presents challenges such as randomness in generated outputs when using machine learning techniques, verification of the generated test cases, etc. Arcuri et al. [12] discussed such challenges of automated test case generation and resolution. Gutierrez-Madronalput et al. [13] put a special focus on information while approaching IoT test case generation, as IoT systems are required to process vast bundles of information in real-time. The authors proposed IoT-TEG, a general structure that can generate a series of scenarios for testing and evaluating IoT systems' performance. In addition, the system can create events with specific values. Barigga et al. [14] proposed a model-driven development approach to specify, produce, and implement the simulation of IoT systems. This method may create and deploy sophisticated IoT simulation environments without coding. While dealing with technical specifications for an IoT project, one of the major prerequisites for a model is to recognize and track entities throughout the specification. A new approach called SOLIMVA is proposed by Santiago Júnior et al. [15]. Its goal is to generate test cases based on models while considering deliverables related to natural language needs. These deliverables have issues with consistency, incompleteness, ambiguity, and poor understandability. Kim et al. [16] evaluated to what extent a language model can determine the final state of an entity after being provided with a description of the initial state and the subsequent operations. Mala et al. [17] suggest using Unified Markup Language (UML) to represent IoT systems as state machines and diagrammatic test case generation. The paper evaluates the meaningfulness of opting for a diagrammatic approach.

Olianas et al. [18] focus on safety-critical situations to address the crucial challenge of assuring the quality and dependability of Internet of Things (IoT) systems. It suggests using the “Matter” tool, which is intended for end-to-end (E2E) testing of IoT systems in a semi-automated fashion. The study describes the difficulties of testing IoT systems, such as the diversity of platforms, resource constraints, data rate, and machine learning components.

Since the advent of the Internet of Things, numerous studies have focused on various innovative methods and approaches to enhance IoT quality assurance and develop effective testing frameworks. However, an unexplored avenue remains the integration of language models. Despite the widely acknowledged importance of IoT testing, only some studies [19] are devoted to evaluating the usefulness and viability of language models in this setting. This paper highlights this gap by investigating the potential benefits and challenges of leveraging state-of-the-art language models for IoT test case generation. This work explores the relationship between language models and IoT testing and how a framework can be developed that can help numerous industries develop secure, reliable, and fail-proof systems.

Language models have proven to be highly effective in parsing the structure and context of a technical document by accurately identifying the underlying entities and relationships. In addition, language models are good at tracking the state of the entities within a specified workflow, which is crucial for describing electronic systems. Moreover, it can help to generate state machine diagrams through Unified Markup Language (UML). UML diagrams are a significant focus for our proposed solution since these diagrams can represent data flow, control flow, and functionalities effectively. The motivation for this paper is to leverage this technology and contribute to the research landscape of IoT system design and testing. The TCG-IoT framework incorporates state-of-the-art advances in natural language processing (NLP) by employing large language models (LLMs) such as Llama 2 and Code Llama [20]. These models are fine-tuned or prompted to understand and extract structured meaning from technical documentation, requirements, and IoT system specifications. Using RAG, the framework augments the input context with domain-specific knowledge, which enables the LLM [21] to generate test cases that are:

• Semantically accurate: The test case logic aligns with the described requirement.
• Consistently formatted: Each test case follows a template (ID, description, expected result, steps).
• Code-compliant: Using Code Llama, the framework generates C test scripts that follow language syntax, naming conventions, and best practices used in embedded IoT systems.

The paper focuses on contributing meaningfully to the IoT system design and testing by:

• Exploring the relationship between state-of-the-art language models and IoT testing
• Developing a practical framework to deliver textual and diagrammatic outputs to highlight bottlenecks, pain points, data flow, etc.
• Generate test cases that consider the technical specifications and ensure the system is tested end-to-end.
• Describing a framework that will consider a range of IoT devices across different categories.

## Method details

This section outlines the architecture and operational flow of the proposed automated testing framework, TCG-IoT, designed for smart home automation systems. The goal is to generate and validate comprehensive software—and hardware—oriented test cases based on the system specifications, leveraging cloud-based language models and a technical knowledge base.

### Components of the system

**Fig. 1:**
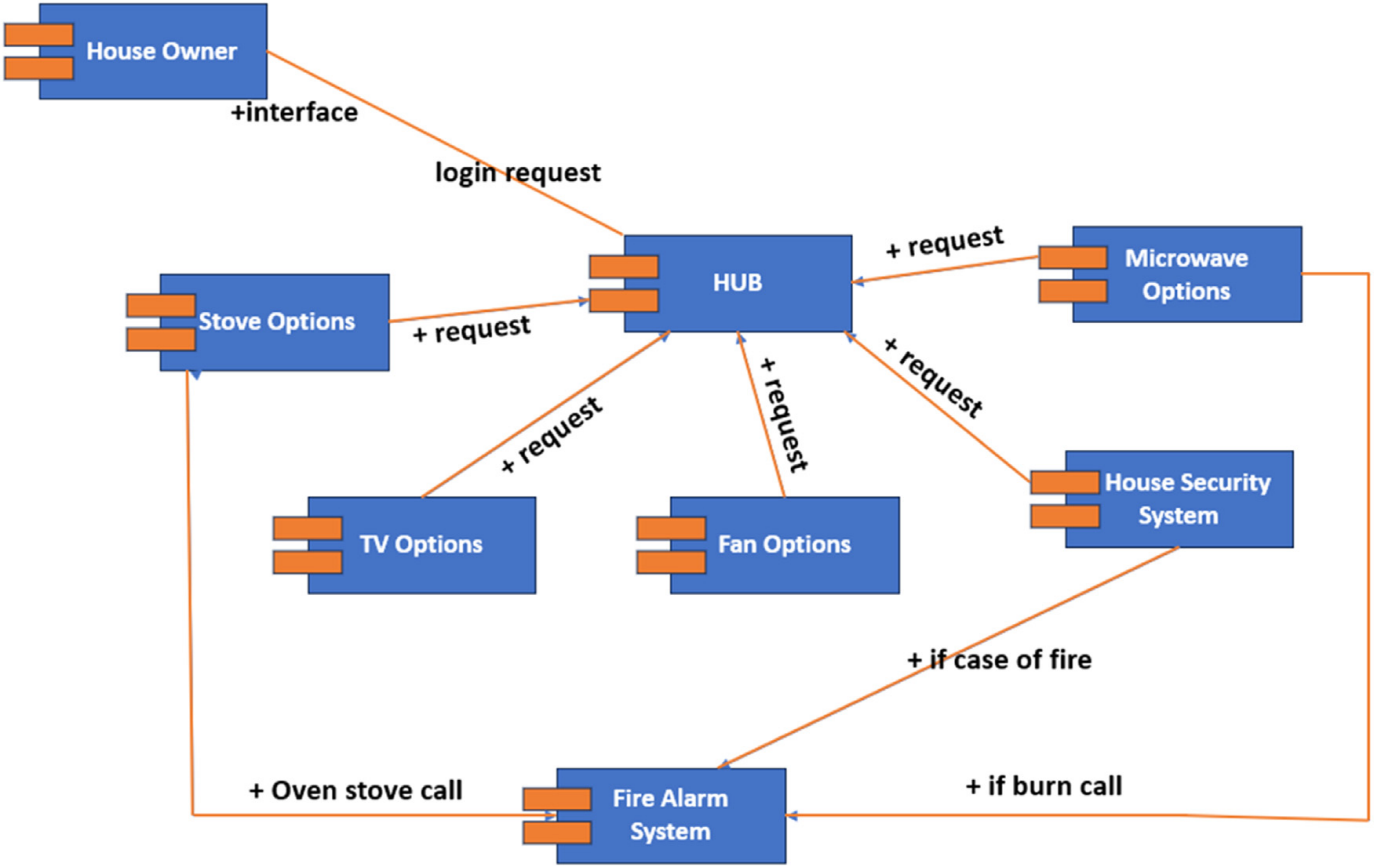
Component of smart home automation system.

The objective of this system [22] is to maintain a hub-based intranet application that enables the house owner to operate appliances and devices in the house without physical presence while also protecting the house against security breaches or fire alarms. The system's users include the house owner, hub, cloud database system, fire department, and Police Department. Fig. 1 describes the arrangement of the components of the smart home automation system.

The components of this system are:

• Owner: The users have a login screen where the authorized administrator/user will log in to the system using a username and password. He/she can add or remove functions from the hub and disconnect devices or connect old devices on the hub. The data is stored in a cloud database. The administrator has access to this database and can access the function present in the database.
• Hub: When the user requests to access the monitoring system or any sensor detects some unusual reading, it requests to alert the user. All these information transfers and requests are done through the hub.
• Appliances: The hub can monitor and access multiple household appliances. These appliances include fans, air conditioners, refrigerators, microwaves, televisions, ovens, home security systems, sensors, and more.
• Sensor: Different sensors are used to monitor different activities in the house. Temperature sensors are used to monitor and maintain the set temperature in the house using fans or air conditioning. Motion sensors detect physical activity; when set on alert, any motion detected will alert the user and connect him to the live camera feed. Smoke detectors alert the fire department and the user if they detect something burning.

**Fig. 2:**
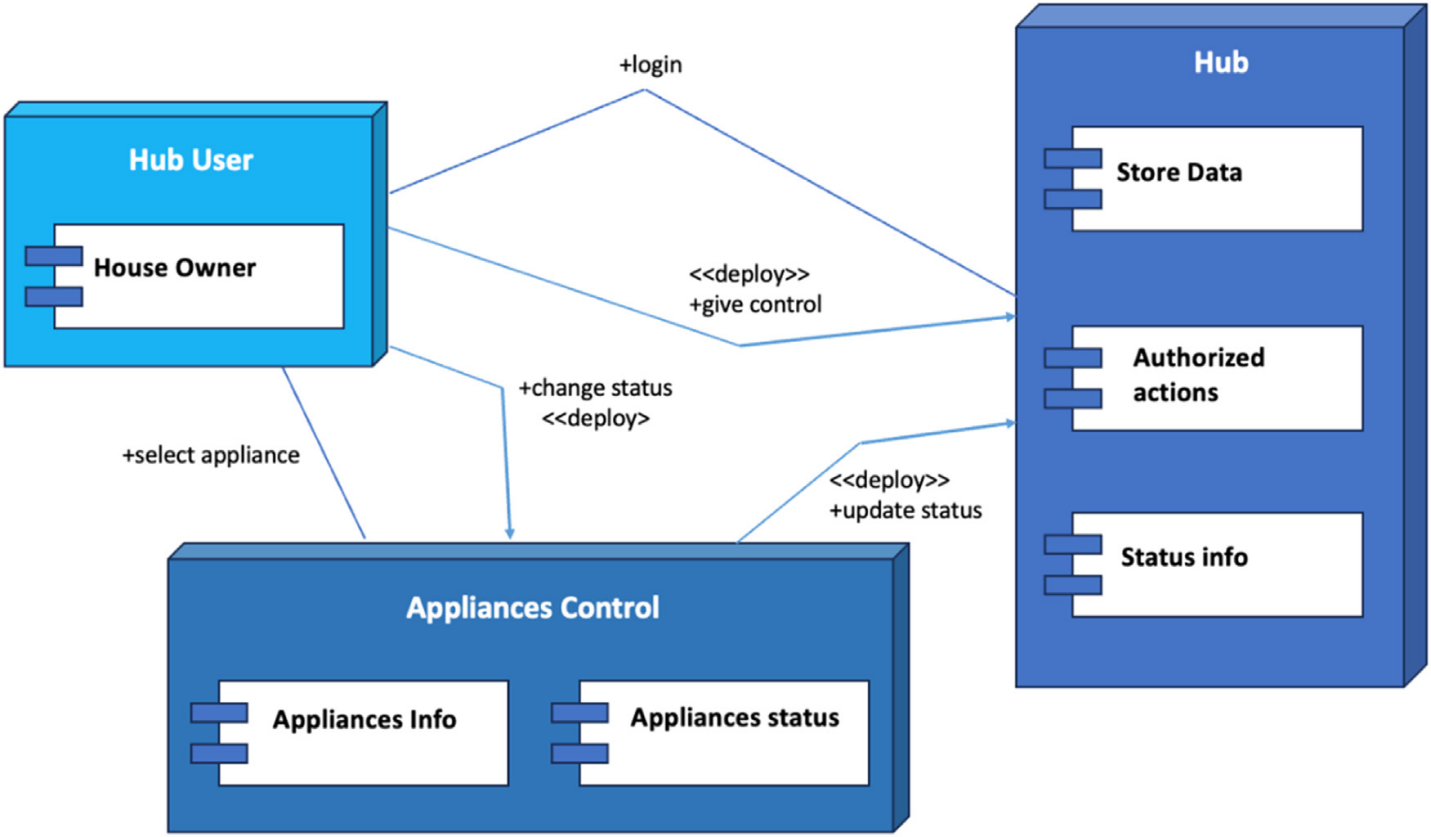
Deployment diagram of smart home automation system.

Fig. 2 depicts the deployment diagram of the smart automation system. The hub is the central controller, typically located at the home's central location.

This location should have good Wi-Fi coverage and easy access for maintenance and configuration. The user data is usually stored on the cloud, hub, or individual smart devices. The data stored on the cloud can be easily accessed using the cloud. The data stored locally on the hub have a faster response and can work in offline conditions. The sensors are placed physically close to the appliance to be monitored to get accurate information. Technical specifications for the IoT system include requirements for the hub or controller, user log in and authentication, various sensors, security controls, and wireless and wired connectivity. The hub/controller should have a quad-core processor, at least 2GB of RAM, 8GB of storage, and support Wi-Fi, Ethernet, and Zigbee/Z-Wave for device communication. User authentication includes password-based, two-factor (2FA), and biometric authentication. Temperature sensors provide accurate readings within −40 °C to 85 °C, with real-time data reporting. Motion sensors have an adjustable detection range of up to 30 feet, a field of view of 90–180 degrees, and real-time detection notifications. Smoke detection and fire alarm systems feature photoelectric or ionization smoke detectors, loud alarms, and immediate user alerts. Security controls offer smartphone or voice-activated smart locks, high-definition security cameras, alarms, and secure access control with encryption. Smoke and CO sensors use different sensor types and have extensive coverage, loud alarms, and multiple power and connectivity options. Water leak sensors employ water-sensing probes, offer audible alarms, and extend battery life. Light sensors measure Lux or foot candles, provide accurate readings, and integrate with smart lighting devices. For wireless connectivity, 802.11ac or 802.11ax Wi-Fi is recommended for high-speed, reliable connections. Zigbee or Z-Wave is essential for smart device communication, forming robust mesh networks. Wired connectivity, including Ethernet ports, ensures stability. Security measures incorporate WPA3 encryption, firewalls, and intrusion detection for enhanced network protection.

**Fig. 3:**
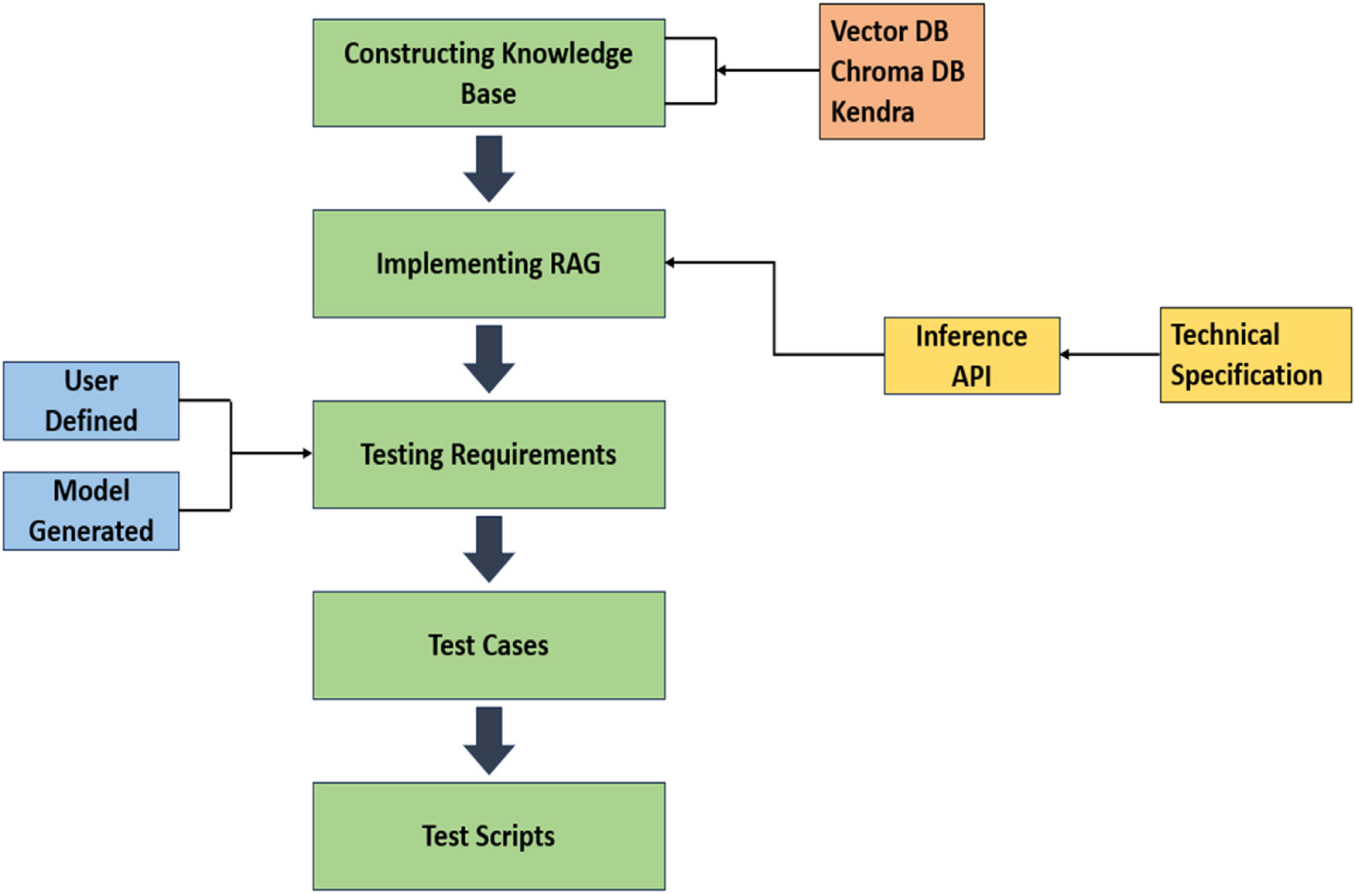
Architecture of testing framework.

Fig. 3 presents a process flow for a testing framework that utilizes the Llama 2 language model developed by Meta, which is deployed on the cloud using AWS SageMaker. The framework aims to enhance IoT testing principles and practices by incorporating a knowledge base of technical literature. This is achieved by constructing a vector database that contains relevant information for IoT testing.

### System overview

The architecture (Fig. 3) integrates a cloud-hosted Llama 2 language model (via AWS SageMaker) with a vector database (e.g., AWS Kendra or ChromaDB) populated from a curated technical knowledge base. This combination supports intelligent, context-aware test case generation using Retrieval Augmented Generation (RAG).

### Step-by-Step framework operation

• **Knowledge Base Construction:** The constructed knowledge base, which comprises various technical documents detailing the best practices and design principles of IoT testing, is uploaded to an AWS S3 bucket. This S3 bucket is now connected to a Vector DB platform such as AWS Kendra or Chroma DB. This allows for querying the documents based on the user prompt within the AWS pipeline, abstracting this process from the cloud.
• **Requirement Acquisition:** Since the proposed framework adopts the V-Model of testing, it is crucial to define the requirements first. The user can upload a CSV file (A simple file format used to store tabular data, where values are separated by commas. In our framework, it is used for importing and exporting test case specifications.) containing a set of testing requirements, which will, in turn, dictate the kind of test cases generated in further steps. The user may need a predefined set of requirements at that stage. Users can query the language model using a simple prompt: “Generate a set of test case requirements for this [system].” The model will go through the constructed knowledge base, extract the relevant points, and then formulate requirements for the particular project. Ensuring the knowledge base has the appropriate documents containing technical details about developing test case requirements.
• **Retrieval-Augmented Querying:** The framework employs the Retrieval Augmented Generation (RAG) approach in this work. RAG queries the vector database to retrieve chunks of information that can supplement the language model. By integrating this contextual information, the framework generates test cases that are accurate and relevant to the specific context.
• **Test Case Generation:** After a set of requirements is established, the next step involves generating the test cases. Through RAG, the framework formulates a set of test cases. Maximum coverage of the IoT system must be ensured. In addition to test cases, the framework generates a series of steps to perform the test case and a few evaluation metrics to judge the system's performance in that particular test case. The generated test cases are organized into four categories: failing criteria, efficiency criteria, bottlenecks, and data flow. Each test case includes a unique test case name, as well as clear and concise steps for performing the test. Additionally, evaluation metrics are provided to measure the effectiveness and adequacy of the test cases.
• **Test Script Generation:** As seen in the various applications developed for software testing using language models, it is found that LLMs can write code, particularly well in high-level languages like Java or Python. It is known that C/*C*++ is one of the most used programming languages for embedded and IoT systems. The model must generate C/*C*++ code for the components that can be tested using code. Backend services of an IoT system need to be tested effectively, as these components are responsible for data management, traffic handling, device configuration, etc. These services can be tested using code. This paper deals with generating actual test scripts for software-based test cases and delivering a series of steps to perform the other hardware-oriented test cases. For understanding and writing code, there is a requirement for focused language models that are fine-tuned for these purposes. Belonging to the family of language models released by Meta, Code Llama is an AI model built on Llama 2, fine-tuned for generating and discussing code. It's available for free for research and commercial use. This remarkable technology can create code and explanations for code, whether you input code or use plain language prompts. It's also useful for code completion and debugging. It supports numerous popular programming languages today, including Python, *C*++, Java, PHP, Typescript (akin to JavaScript), Bash, and more.
• **Output Formatting:** The final outputs include: a .csv file containing structured test requirements and test cases .c/.cpp scripts for software tests. These can be directly imported into test automation pipelines or analyzed independently.
• **Validation Criteria:** The generated test cases are validated based on: [TC1] Comprehensive coverage of hardware/software components, [TC2] Conformance to the V-Model of system testing, [TC3] Fulfillment of verification and validation (V&V) principles.

### Pseudocode for the proposed framework

**Fig. 4:**
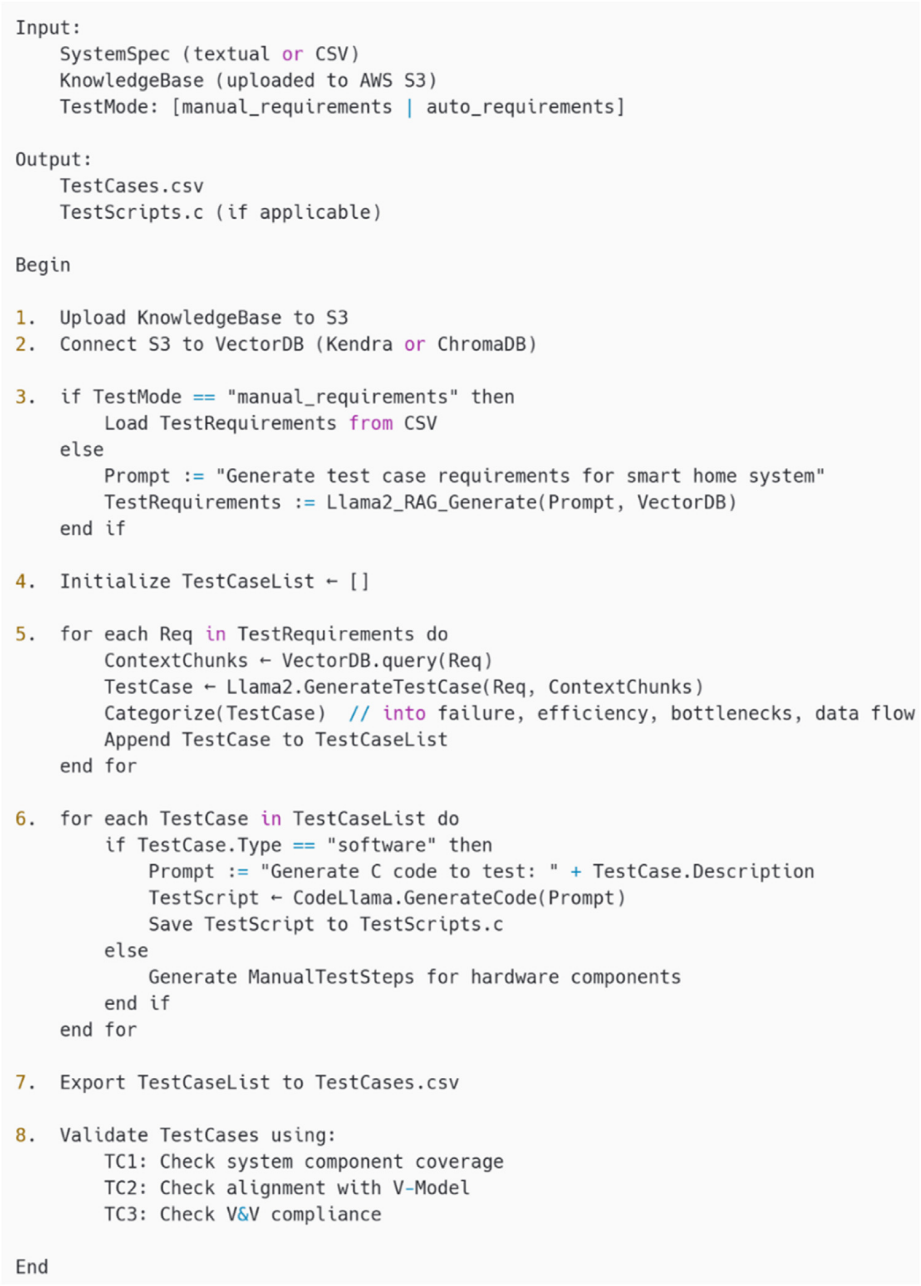
Pseudo code for the proposed framework.

TG-IoT automates the generation of test cases and scripts for smart home IoT systems using Llama 2 and Code Llama models. As shown in the pseudo-code of TCG-ToT in Fig. 4, It integrates user-defined or auto-generated requirements with a knowledge base queried via Retrieval Augmented Generation (RAG) to ensure comprehensive, V-Model-aligned testing of software and hardware components.

## Method validation

In this section, the work discusses the step-by-step process of using the proposed testing framework and evaluates the results generated. The article considers one case study of IoT systems, i.e., smart home automation. These systems pose many technical complexities, which the framework aims to simplify. This section discusses both systems in detail and describes integrating the testing framework into such systems. The generated test cases and test scripts are evaluated on the following testing criteria (TC):

• [TC 1] Maximum coverage of the systems is ensured.
• [TC 2] The generated test cases meet the defined requirements as per the V-Model.
• [TC 3] Validation and Verification, i.e., to determine if a system or component satisfies its operational and system-level requirements

As per the V-Model, defining the requirements beforehand is the key element in software testing. These requirements dictate the type of test cases that will be generated. As per the validation and verification model, there must be a precise mapping between the pre-defined requirements and the generated test cases. The proposed framework allows users to input their defined requirements for a given IoT system. It provides the flexibility to create the test case requirements using the model. The model reads and comprehends the technical specifications of the smart home automation system in this case and generates a set of related testing requirements. The output is customized in CSV format for better readability and understanding. The generated testing requirements can be seen in Table 1.

**Table 1:** Testing requirement.

Test Case ID	Description	Expected Results
TC001	Hub/Controller Processor Quad-core Requirement	The processor is quad-core with detailed specifications that match the requirements.
TC002	Hub/Controller RAM Requirement	Hub/controller RAM is 2GB or more as specified.
TC003	Hub/Controller Storage Capacity Requirement	As stated, the Hub/controller has at least 8GB of storage capacity.
TC004	Hub/Controller Connectivity Requirements	Hub/controller supports Wi-Fi (802.11n/ac), Ethernet, Zigbee, and Z-Wave for device communication.
TC005	User Authentication Methods Requirement	User authentication includes password-based, 2FA, and biometric authentication options where applicable.
TC006	Temperature Sensor Accuracy Requirement	Temperature sensor provides high-precision, real-time temperature readings within the −40 °C to 85 °C range.
TC007	Motion Sensor Detection Range Requirement	Motion sensors allow for an adjustable detection range of up to 30 feet with FOV in the 90–180 degrees range and provide real-time detection notifications.
TC008	Smoke Detector and Fire Alarm Types Requirement	Smoke detectors include photoelectric and ionization types, feature loud alarms with adjustable volume, and provide immediate user alerts.

Based on the requirements given by the user or the requirements generated within the framework, the model implements the RAG mechanism and queries the technical literature for testing design practices and norms. The relevant query results retrieved from the vector database dictate the generation of test cases for a given IoT system. The test cases generated by TCG-IoT for a smart home automation system can be seen in Table 2.

**Table 2:** Generated testing cases.

Test Case ID	Description	Expected Results
TC001	Hub/Controller Processor Quad-core Verification	The hub/controller device powers on successfully. The documentation provides processor specifications indicating that the processor is quad-core.
TC002	Hub/Controller RAM Capacity Verification	The hub/controller device powers on successfully. The documentation provides RAM specifications indicating that the RAM capacity is 2GB or more, as specified.
TC003	Hub/Controller Storage Capacity Verification	The hub/controller device powers on successfully. As stated, the documentation provides storage capacity specifications indicating that the device has at least 8GB of storage capacity.
TC004	Hub/Controller Connectivity Verification	The hub/controller device powers on successfully. The device supports Wi-Fi (802.11n/ac) and Ethernet connectivity. Compatibility with Zigbee and Z-Wave for device communication is verified.
TC005	User Authentication Methods Verification	The hub/controller device powers on successfully. User authentication methods include password-based, 2FA, and biometric authentication options where applicable.

Table 3 presents sample Retrieval-Augmented Generation (RAG) queries used to generate test case requirements for a smart home system. Each query is processed by the LLM using retrieved technical context, followed by test case generation and evaluation using relevance scores and manual validation. The LLM usage in all instances is prompt-based, without any domain-specific fine-tuning. This illustrates how RAG effectively bridges natural language prompts with technical testing standards in a flexible and adaptable manner.

**Table 3:** Example RAG query, output, and metrics.

Test Case ID	Description	Example RAG Query & Retrieved Context	Generated Output	Metrics	LLM Usage (Prompt/Fine-tuned)
TC001	Hub/Controller Processor Quad-core Requirement	Query: “What is the recommended processor spec for a smart home hub?” Retrieved: “Quad-core processors provide parallelism and responsiveness in IoT systems.”	Documentation confirms that the hub/controller has a quad-core processor that matches the system specs.	Relevance: 93% Manual Review: Passed	Prompt-based
TC002	Hub/Controller RAM Requirement	Query: “Is 2 GB RAM sufficient for a home automation controller?” Retrieved: “Minimum 2GB RAM is recommended for handling multiple smart devices simultaneously.”	Validate that the hub/controller has ≥ 2GB RAM as per system spec.	Relevance: 91% Manual Review: Passed	Prompt-based
TC003	Hub/Controller Storage Capacity Requirement	Query: “Minimum storage required for IoT hub?” Retrieved: “At least 8GB flash is needed for firmware and device configs.”	Ensure the hub/controller has ≥ 8GB storage.	Relevance: 90 % Manual Review: Passed	Prompt-based
TC004	Hub/Controller Connectivity Requirements	Query: “Which protocols are used in smart home hubs?” Retrieved: “Wi-Fi (802.11n/ac), Zigbee, Z-Wave, and Ethernet are common for interoperability.”	Check whether the device supports Wi-Fi, Ethernet, Zigbee, and Z-Wave.	Relevance: 94 % Manual Review: Passed	Prompt-based
TC005	User Authentication Methods Requirement	Query: “What authentication should a smart home system support?” Retrieved: “Password, two-factor authentication, and biometrics enhance system security.”	Confirm that the hub provides password, 2FA, and biometric options where applicable.	Relevance: 95 % Manual Review: Passed	Prompt-based

For test cases generated for the hardware components, the TCG-IoT framework generates a series of steps to perform the tests and evaluation metrics to judge the system's performance in each test case. For software components such as backend services and data storage/retrieval, the framework generates test case code using the Code-Llama [23] language model. The code generated is in the C programming language, a low-level language widely used in IoT and embedded systems development.

Table 4 compares the proposed work with the current related work by comparing their similarities and differences. The comparison highlights the novelty of the proposed work. It sheds light on possible future improvements, such as connecting the executable test scripts to simulation and testing environments, considering diagrammatic representation such as UML, etc.

**Table 4:** Comparison of similarities and differences between proposed and related work.

Reference	Similarities	Advantages of TCG-IoT (proposed method)
**MATTER** [26]	End-to-end automated test case generation	MATTER uses model-based testing, while TCG-IoT uses language models. TCG-IoT generates direct output test scripts in C without considering simulation environments.
**IoT-TEG** [13]	Considers requirements to generate events	IoT-TEG is a test event generator system with values to test functionalities, while TCG-IoT generates testing requirements, test cases, steps to perform, and test scripts.
**Model-Based Automatic Test Case Generation** [24]	Automatic test case generation for both software and hardware components	Test case generation is done through a UML model, whereas TCG-IoT does not consider diagrammatic representations.
**Towards an Automated Testing Framework for IoT Devices** [25]	Test case generation	Automatic client frameworks are developed in Python and connected to testing environments. In contrast, TCG-IoT outputs are in C and not linked to testing or simulation environments.

## Discussion

The TCG-IoT framework enables comprehensive test case generation by manually extracting or accepting input system specifications (via CSV) or automatically through NLP-based parsing. These specifications define the core requirements of the IoT system. Each requirement is converted into a semantic query to a vector database built from IoT testing literature using the RAG mechanism. This database provides relevant contextual information that supplements the language model's knowledge and guides it in generating system-specific test cases. The process ensures that each component of the IoT system (e.g., processor, RAM, connectivity protocols, sensors, authentication methods) is mapped to one or more test cases. It also ensures best practices and technical standards retrieved from literature are incorporated into each test’s context. High coverage is maintained through requirement-to-test mapping validated against the V-Model. While TCG-IoT successfully automates the generation of test cases using prompt-based language models, particular security and reliability concerns must be addressed. LLM-generated outputs may occasionally contain:

• Hallucinations or inaccurate suggestions that do not align with system specifications.
• Incomplete validation logic if the prompt lacks clarity or insufficient retrieved context.
• Security blind spots, especially when the documentation does not explicitly state authentication or encryption details.

To mitigate these, the framework currently supports:

• Manual review checkpoints, as shown in Table 3, where each generated test case is verified by an engineer.
• Prompt tuning using domain-specific language.
• Integration of static analysis and verification tools in future versions to validate the generated C code against formal specifications.

The TCG-IoT framework is designed with scalability in mind. The system supports dynamic processing of large technical specifications and device catalogs by leveraging cloud-hosted LLMs (such as Llama 2 deployed via AWS SageMaker) and retrieval-augmented generation (RAG). Whether deployed for small-scale smart homes or enterprise-grade IoT infrastructures (e.g., smart cities, industrial automation), the modular structure allows the framework to process multiple requirements sets in parallel, generate tailored test cases per subsystem, and scale its testing capabilities as system complexity grows. Furthermore, integrating with vector databases like ChromaDB enables efficient retrieval of domain-specific knowledge at scale, enhancing accuracy while maintaining performance. The TCG-IoT framework goes beyond basic automation by ensuring that each test case generated is contextually informed by a rich corpus of IoT testing practices. Through the Retrieval-Augmented Generation (RAG) mechanism, the framework retrieves relevant technical details from the vector database. It uses them to guide the LLM in generating highly relevant and system-specific test cases. This process:

• Ensures maximum system coverage by accounting for diverse components like sensors, connectivity modules, authentication protocols, and controllers.
• Aligns generated test cases with predefined requirements and V-Model-based verification principles.
• Increases reliability by ensuring that each test case is backed by industry best practices and verified specifications, reducing ambiguity or omissions in testing.

In the Smart Home Automation case study, TCG-IoT was able to cover various test categories such as performance (RAM, processor), connectivity (Zigbee, Wi-Fi), safety (fire sensors), and security (authentication methods). All test cases were automatically validated against the expected outcomes using contextual relevance metrics and manual review, showcasing the practical reliability of the approach.

## Conclusion

The increasing IoT and electronic systems adoption requires robust real-time data processing and management solutions. However, their inherent complexity poses significant challenges in software and hardware testing. Traditional hardware testing is often manual, inefficient, and resource-intensive. This work introduces TCG-IoT, an automated testing framework that bridges the gap between IoT and software testing by leveraging NLP-based language models. This paper explores smart home automation in a case study to validate the framework's capabilities. Technical specifications for these systems were input into the framework, which utilizes the Retrieval-Augmented Generation (RAG) mechanism to query a knowledge base consisting of IoT testing best practices and design patterns. Inspired by the V-Model in software testing, the framework ensures that all generated test cases directly map to predefined requirements, covering all essential testing criteria (TC1, TC2, TC3). By automating software and hardware testing, TCG-IoT provides detailed test execution steps for hardware components and generates test scripts in C using the Code-Llama language model. The framework successfully delivers a comprehensive suite of testing requirements, test cases, and scripts, enabling users to assess IoT systems' feasibility, limitations, and bottlenecks before finalizing software and hardware development. The main contribution of this research lies in integrating LLMs with RAG to create domain-specific, verifiable, and context-aware test artifacts, significantly improving test coverage, traceability, and cost-effectiveness in IoT testing. The practical implications of this framework include reducing manual effort, increasing test reliability, and offering a scalable solution for industry-grade IoT deployments where test generation must adapt to dynamically changing specifications. For future work, the natural language processing capabilities and task-specific adaptability of large language models (LLMs) can be extended to integrate simulation-based testing. The simulation will enable real-time performance evaluation of IoT systems against generated test cases. However, simulating IoT environments presents challenges, such as the need for complex execution environments, specialized SDKs, and support for diverse communication protocols like MQTT. In addition, future studies could explore integrating visual-based prompts (e.g., UML diagrams or hardware schematics), supporting multilingual document ingestion, and coupling the framework with reinforcement learning for continuous test optimization. Overall, TCG-IoT offers a novel and promising approach to modernizing and automating IoT testing practices in line with next-generation industry demands.

## Limitations

Not applicable.

## Ethics statements

This research did not involve research on humans or animals, and no data is involved from social media platforms.

## CRediT authorship contribution statement

**Sumit Kumar:** Writing – original draft, Validation, Visualization, Methodology. **Kiran Napte:** Writing – review & editing, Funding acquisition. **Ruchi Rani:** Supervision, Writing – review & editing. **Sanjeev Kumar Pippal:** Supervision, Writing – review & editing.

## Declaration of competing interests

The authors declare that they have no known competing financial interests or personal relationships that could have appeared to influence the work reported in this paper.

## Data Availability

No data was used for the research described in the article.
